# Revealing the Role of High-Density Lipoprotein in Colorectal Cancer

**DOI:** 10.3390/ijms22073352

**Published:** 2021-03-25

**Authors:** Aleksandra Zeljkovic, Jelena Vekic, Marija Mihajlovic, Tamara Gojkovic, Sandra Vladimirov, Dejan Zeljkovic, Vesna Spasojevic-Kalimanovska, Bratislav Trifunovic

**Affiliations:** 1 Department of Medical Biochemistry, Faculty of Pharmacy, University of Belgrade, 11000 Belgrade, Serbia; jelena.vekic@pharmacy.bg.ac.rs (J.V.); marija.mihajlovic@pharmacy.bg.ac.rs (M.M.); tamara.gojkovic@pharmacy.bg.ac.rs (T.G.); sandra_vladimirov@yahoo.com (S.V.); vkalima@pharmacy.bg.ac.rs (V.S.-K.); 2 Clinic of General Surgery, Military Medical Academy, 11000 Belgrade, Serbia; delezeljkov@gmail.com (D.Z.); mavumit@gmail.com (B.T.); 3 Faculty of Medicine of the Military Medical Academy, University of Defense, 11000 Belgrade, Serbia

**Keywords:** colorectal cancer, high-density lipoprotein, HDL particle distribution, HDL functional properties, genetic background, cholesterol trafficking, therapeutic implications

## Abstract

Colorectal cancer (CRC) is a highly prevalent malignancy with multifactorial etiology, which includes metabolic alterations as contributors to disease development. Studies have shown that lipid status disorders are involved in colorectal carcinogenesis. In line with this, previous studies have also suggested that the serum high-density lipoprotein cholesterol (HDL-C) level decreases in patients with CRC, but more recently, the focus of investigations has shifted toward the exploration of qualitative properties of HDL in this malignancy. Herein, a comprehensive overview of available evidences regarding the putative role of HDL in CRC will be presented. We will analyze existing findings regarding alterations of HDL-C levels but also HDL particle structure and distribution in CRC. In addition, changes in HDL functionality in this malignancy will be discussed. Moreover, we will focus on the genetic regulation of HDL metabolism, as well as the involvement of HDL in disturbances of cholesterol trafficking in CRC. Finally, possible therapeutic implications related to HDL will be presented. Given the available evidence, future studies are needed to resolve all raised issues concerning the suggested protective role of HDL in CRC, its presumed function as a biomarker, and eventual therapeutic approaches based on HDL.

## 1. Introduction

Colorectal cancer (CRC) is among the most prevalent malignant diseases in the modern world [1]. CRC has a complex etiology, and both genetic and environmental factors participate in its development [2]. Metabolic alterations are recognized as important contributors to colorectal carcinogenesis; among them, obesity and insulin resistance are firmly established as significant risk factors for CRC development [3]. An inevitable feature of obesity-related metabolic complications is dyslipidemia. Therefore, numerous studies have investigated the role of lipid alterations in the etiopathogenesis of CRC.

Among other lipid status irregularities, CRC patients experience changes in high-density lipoprotein cholesterol (HDL-C) levels [4]. However, HDL is, by far, the most complex lipoprotein particle [5], and it is now clear that its role both in health and in disease goes beyond reverse cholesterol transport (RCT) and includes anti-oxidant, anti-inflammatory, immunomodulatory, anti-apoptotic, and anti-thrombotic functions [6,7,8]. The most extensive studies of HDL were performed with respect to its role in the prevention of atherosclerosis development. Nowadays, it is firmly established that thanks to its complex protein and lipid structure, HDL possesses numerous atheroprotective properties. A fundamental contribution of HDL to the prevention and delaying of atherosclerosis development is its role in RCT [9]. Namely, HDL promotes the efflux of cholesterol from foam cells, thus reducing cholesterol accumulation in atherosclerotic lesions. Today, it is accepted that apart from the well-known ATP-binding cassette transporter A1 (ABCA1)-mediated efflux, at least three additional transport mechanisms are involved in cholesterol uptake by HDL [10,11]. Furthermore, HDL participates in maintaining the redox balance by reducing low-density lipoprotein (LDL) oxidation and thereby preventing detrimental pro-atherosclerotic effects of LDL in the subendothelial space [12]. HDL also stimulates the production of vasodilatory and anti-aggregative nitric oxide (NO) [12]. An additional atheroprotective property is reflected through the capability of HDL to inhibit endothelial cell apoptosis via modulation of the expression of pro-apoptotic and anti-apoptotic proteins [9]. Last, but certainly not the least, HDL performs anti-inflammatory and immunomodulatory activities. This lipoprotein prevents the conversion of macrophages to the pro-inflammatory M1 phenotype [13], thus decreasing the pro-inflammatory milieu typical for atherosclerosis development. Studies have shown that functional microRNAs are delivered to target cells by HDL, and such HDL-mediated transport contributes to anti-adhesive and anti-inflammatory effects in the endothelium [9,14]. Although the roles of HDL in atherosclerosis and related conditions are well described, there is a lack of data regarding the potential involvement of HDL in the onset and progression of other systemic diseases, including CRC. Yet, keeping in mind the close association of CRC with obesity and metabolic changes, HDL functions should be evaluated in this type of malignancy. In this review paper, we will analyze recent evidence regarding changes in the structural and functional integrity of HDL in CRC. We will focus on HDL-C levels, the composition of HDL particles, different aspects of its functionality, and its interactions with receptors and other regulatory proteins in colorectal malignancies. In addition, research findings of genetic alterations that are linked to HDL in CRC will be summarized. Finally, we will address the issue of the potential benefit of HDL-based therapeutic procedures in this disease.

## 2. HDL-C Levels and HDL Subclass Distribution in CRC

**Figure 1:**
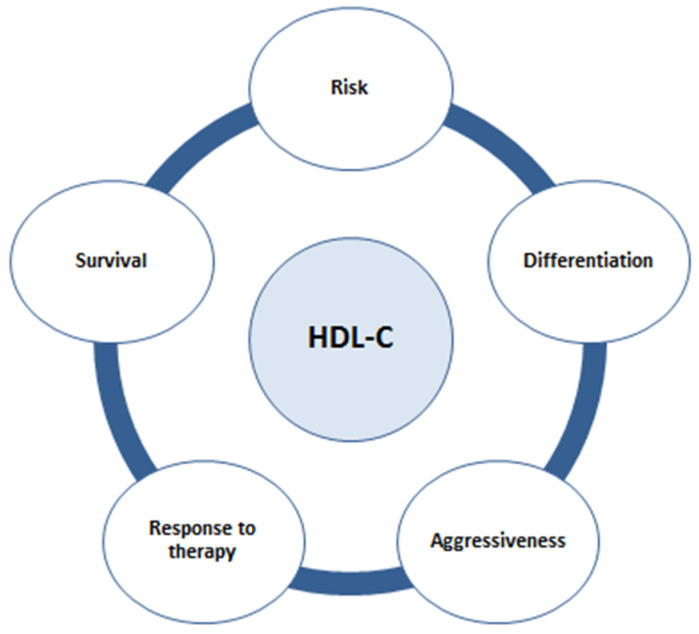
The relationship between high-density lipoprotein cholesterol (HDL-C) levels and colorectal cancer (CRC).

A reduced serum concentration of HDL-C was observed in patients with different types of cancers [15]. According to a meta-analysis of randomized controlled trials of lipid-altering interventions, every 10 mg/dL increase in the HDL-C level reduces the risk of cancer by 36% [16]. Although these data suggest that HDL particles might play a protective role against cancer development, the relationship between HDL-C levels, cancer incidence, and prognosis is not fully elucidated and seems to be influenced by the type of tumor [15]. In this regard, accumulating evidence suggests that a higher HDL-C level is associated with both a decreased risk of CRC development and increased survival (Figure 1). Of note, generalizability of the results of available studies is limited due to the small sample size and incomplete covariate information, since some of the studies were not specifically designed for lipid analyses. The European Prospective Investigation into Cancer and Nutrition (EPIC) study [17], which included cohorts from 23 centers in 10 European countries, found an inverse association between HDL-C and apolipoprotein AI (apoAI) levels with the risk of colon cancer. In addition, a meta-analysis by Yao et al. [18] confirmed that a higher HDL-C level is associated with a decreased risk of CRC.

The results of several studies suggest an intriguing association between HDL-C levels and tumor incidence at specific anatomical sites within the colorectum. For instance, the results of the EPIC study imply that the link between HDL-C levels and cancer risk has a higher significance in subjects with colon cancer than in those with rectum cancer [17]. According to the results of Zhang et al. [19], serum HDL-C levels in patients with rectum cancer are significantly lower than the levels in patients with colon cancer. A study of patients with gastrointestinal, colon, and rectal cancer undergoing surgical treatment showed that patients with invasive and poorly differentiated tumors (grades III and IV) had lower serum HDL-C levels than those with non-invasive, moderately differentiated tumors (grades I and II) [20]. Zhang et al. [19] demonstrated significantly higher HDL-C levels in cancerous than in paracancerous tissue of patients with CRC. Moreover, the level of HDL-C in the tissue of CRC patients at tumor-node-metastasis (TNM) stages III and IV was significantly higher than in patients at TNM stages I and II [19]. In a study by Healy et al. [21], the serum HDL-C level was significantly associated with a more advanced pathological stage of CRC and lymph node involvement. Other studies have found no difference in HDL-C levels among CRC patients with and without distant metastases [22], or even increased HDL-C levels in those with ocular metastases [23].

Recovery from low HDL-C levels at diagnosis to higher levels after adjuvant chemotherapy has been observed in metastatic CRC patients [24,25]. A retrospective analysis of CRC patients’ lipid profile prior to neoadjuvant therapy revealed that those who did not respond well to the therapy had lower HDL-C levels than patients with evident tumor regression [26]. Currently, k-Ras (KRAS) mutation testing is recommended for CRC patients with advanced metastatic disease in order to predict the resistance to epidermal growth factor receptor (EGFR)-targeting therapy [27]. A recent report by Tabuso et al. [28] showed a significant association of a high serum cholesterol/HDL-C ratio as indicative of decreased HDL-C levels, with the presence of the KRAS mutation in recto-sigmoid cancers, which suggests that the serum lipid profile at diagnosis could be a valuable tool for predicting an individual’s response to therapy.

Low pre-operative HDL-C levels in patients with stage II/III CRC are associated with worse disease-free survival (DFS) and overall survival (OS) [29]. On the other hand, chemotherapy-related elevations in HDL-C levels in patients with non-metastatic CRC correlate with a longer DSF and OS [25]. According to the data of Tabuso et al. [28], in patients with KRAS-mutated recto-sigmoid cancer stage III, a high cholesterol/HDL-C ratio at diagnosis was associated with better survival. In contrast, in a subgroup of patients with KRAS-mutated recto-sigmoid cancer stage IV, a low cholesterol/HDL-C ratio was associated with better survival [28]. The authors used a cutoff 3.5 to discriminate low and high cholesterol/HDL-C ratios, which is appropriate for cardiometabolic diseases but might be less convenient for metastatic CRC. In addition, it is noteworthy that rather a small number of CRC patients with stages III and IV were enrolled in this study; thus these controversial results require further investigation. A study by Hong et al. [30] confirmed that inclusion of the pre-operative lipid profile of patients with non-metastatic CRC improves the prognostic accuracy of a model consisting of routine pathology findings. A recent prospective study showed that increased HDL-C levels have a beneficial impact on recurrence-free survival in CRC patients who have undergone surgical resection [31]. Of note, in the Irish Longitudinal Study on Ageing (TILDA), HDL-C was a unique lipid parameter that was not related to the poor prognosis of patients with CRC after two years of follow-up [32]. In line with the expanding knowledge on the role of HDL in CRC, Huang et al. [33] recently developed a simple prognostic score based on the HDL-C and albumin ratio (HA score). The authors demonstrated a shorter OS and DFS in CRC patients with a high HA score, representing reduced HDL-C and albumin levels [33].

A long-standing hypothesis states that simple determination of plasma HDL-C levels fails to convey all protective functions of HDL particles and to provide information about their remarkable heterogeneity in terms of shape, size, and composition. Besides cholesterol, HDL also carries triglycerides and numerous lipid species of the phospholipid and sphingolipid family, such as various proteins, enzymes, hormones, vitamins, and microRNAs [34]. Hence, it is reasonable to assume that different lipid and non-lipid constituents within HDL might play different roles in tumorigenesis. Phospholipids represent the most abundant lipid species within HDL, followed by cholesterol esters, triglycerides, and free cholesterol. Based on their physicochemical properties, hydrophobic lipids, such as cholesteryl esters and triglycerides, are carried in the core of HDL particles, while amphipathic lipids, i.e., phospholipids and free cholesterol, reside on the surface of HDL [34]. So far, evidence is accumulating that changes in HDL’s lipid composition might affect its anti-atherogenic properties [6]. For instance, triglyceride enrichment and cholesteryl ester depletion of the HDL core alter its anti-oxidative potential [35]. In addition, triglyceride-enriched HDL particles have impaired function within the RCT process [36]. This structural modification is a characteristic feature of insulin resistance, which is regularly seen in patients with obesity, metabolic syndrome, or type 2 diabetes mellitus. Since insulin resistance is recognized as one of the most common links between cardiometabolic and malignant diseases, including CRC [37], it is reasonable to assume that such structural modifications of the HDL core might also be presented in CRC. The pro-inflammatory state is another common characteristic of cardiometabolic diseases and CRC [37], which is able to induce changes in the HDL lipidome. Pruzanski et al. [38] demonstrated significant changes in the lipid composition of HDL under a pro-inflammatory condition, as reflected by a decreased cholesteryl ester/triglyceride ratio in the HDL core and the reduced content of phosphatidylcholine, phosphatidylinositol, and sphingomyelin at the surface of HDL particles. Their study also showed that acute-phase HDL has increased content of lysophosphatidylcholine [38], a biologically active phospholipid involved in the development and progression of various diseases, including cancer [39]. However, very limited data on changes in HDL-associated lipids in malignant diseases are available. In patients with hepatocellular carcinoma, increased plasma phospholipids were found, but the levels of HDL-associated phospholipids did not differ when compared to controls [40]. Evidence from in vitro studies showed that oxidation of HDL can promote breast cancer metastasis, which is attributed, at least in part, to lipid peroxidation within HDL [41]. Since the HDL lipidome in cancer is still largely unexplored, further advances in the research on HDL remodeling in malignant diseases will improve our understanding of the possible impact of these changes on HDL functions during the development of CRC.

Furthermore, assessment of the HDL particle size and subclass distribution could provide prognostic information beyond the HDL-C level, which has been demonstrated in the setting of atherosclerosis [42,43,44]. However, changes in the HDL subclass distribution are seldom investigated in patients with CRC, while the associations of HDL particle characteristics with disease progression and patients’ survival remain completely unknown. In an early study, Dessì et al. [20] evaluated the profile of HDL particles, obtained by HPLC, in a small sample of patients with gastrointestinal cancers, including CRC. The study showed a reduction in the HDL 3 fraction in the serum of patients with gastrointestinal cancer [20]. In our case–control study, CRC patients had a smaller HDL particle size, elevated proportion of small HDL 3b, and reduced proportion of large HDL 2b particles [45]. In addition, both HDL-C level and HDL size constitute an optimal cost-effective model with adequate discriminative abilities for CRC [45]. Recently, Bains et al. [8] performed a small study on post-surgical CRC patients, investigating changes in the HDL subclass distribution during hospitalization. The authors [8] showed a decrease in intermediate and small HDL particles with a concomitant increase in large HDL particles following surgery. It is noteworthy that the main goal of their study was to evaluate the effect of acute inflammation of the HDL subclass distribution [8], so any conclusion regarding the impact of the post-surgical HDL profile on CRC prognosis cannot be drawn. Therefore, future prospective studies are needed to disclose whether the HDL subclass determination has any prognostic potential following surgery or chemotherapy.

## 3. Functional Characteristics of HDL Particles in CRC

As emphasized above, the majority of studies consistently point toward low HDL-C levels in CRC patients. However, there is still a lack of evidence regarding a causal relationship between decreased HDL-C levels and the development of CRC. Moreover, recent Mendelian randomization studies have indicated the possible causal role of circulating levels of total cholesterol but not HDL-C in the etiopathogenesis of CRC [46] or have even ruled out any direct contribution of altered serum lipid concentrations to the onset and progression of the disease [47]. Thus, complex genetic investigations seemingly do not support unequivocal findings of observational studies. Such disagreement urges the need to shift the research focus from HDL-C levels toward qualitative and functional properties of HDL particles.

**Figure 2:**
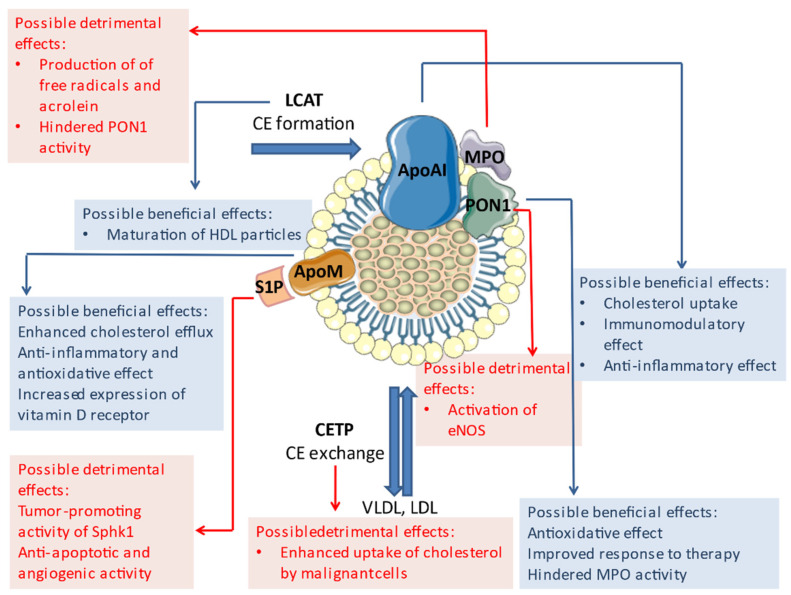
Constituents of HDL functionality and their presumed role in CRC development. ApoAI—apolipoprotein AI; apoM—apolipoprotein M; CE—cholesterol esters; CETP—cholesteryl ester transfer protein; eNOS—endothelial nitric oxide synthase; LCAT—lecithin:cholesterol acyltransferase; LDL—low-density lipoprotein; MPO—myeloperoxidase; PON1—paraoxonase 1; Sphk1—sphingosine kinase 1; VLDL—very low density lipoprotein. The figure was composed using Servier Medical Art templates, licensed under a Creative Common Attribution 3.0 (https://smart.servier.com (accessed on 1 December 2020)).

Nowadays, it is widely accepted that the role of HDL in both physiological and pathophysiological conditions goes far beyond its cholesterol content [48]. Therefore, the possible involvement of HDL in CRC development should be considered in light of its quality and functionality. The term HDL functionality refers to a plethora of known HDL-mediated effects on energy metabolism, the immune system, the inflammatory response, the redox balance, endothelial homeostasis, and various cellular functions [49]. Carriers of HDL functionality are structural components of this lipoprotein, i.e., proteins and complex lipids associated with HDL particles [50,51,52]. Due to the heterogeneity of HDL-conferred functions, it is difficult to develop a unique procedure for precise quantification of the overall functional capacity of HDL. Instead, a variety of assays are used for the assessment of particular HDL functions. The cholesterol efflux capacity is considered a crucial element of HDL functionality [53,54,55], and a range of assays employing radioisotope- or non-radioisotope-labeled cholesterol have been developed for estimation of this process efficacy [52]. In addition, activities of key RCT mediators, lecithin:cholesterol acyltransferase (LCAT) and cholesteryl ester transfer protein (CETP), can be used as indicators of RCT effectiveness [52]. The anti-inflammatory properties of HDL can also be assessed by several methods, which are generally based on co-incubation of different cell types with HDL and measurement of the cellular response to inflammatory stimuli [52,56]. It was proposed that the HDL inflammatory index (calculated as a ratio of the measured effects with and without the presence of HDL in cell culture) could be used as a metric for HDL’s anti-inflammatory properties [57]. Similarly, the anti-oxidative function of HDL can be assessed as a capability of HDL to protect LDL from oxidation [57] or to interfere with the production of reactive oxygen species [52]. Moreover, determination of the activities of HDL-associated enzymes involved in maintaining the redox balance, such as paraoxonase 1 (PON1) and myeloperoxidase (MPO), is also used as a measure of HDL’s anti-oxidative properties [58,59]. The effect of HDL on the expression of endothelial nitric oxide synthase (eNOS) can be assessed by measurement of NO production in the presence of HDL [52], while anti-apoptotic effects can be estimated by the quantification of nucleosomes [51]. Finally, analysis of the HDL proteome and lipidome offers novel possibilities for the quantification of HDL functionality that is reflected through the altered presence of specific lipid or protein constituents [60,61]. HDL contains more than 100 proteins with different functions, but apoAI, apolipoprotein M (apoM), and PON1 are most extensively investigated in terms of their possible role in malignant diseases. In addition, HDL functionality is strongly affected by changes in the activities of LCAT and CETP. Alterations in all these proteins should be considered with respect to colorectal carcinogenesis (Figure 2).

### 3.1. ApoAI

Almost 10 years ago, a study performed within the EPIC study demonstrated that circulating levels of apoAI are inversely associated with the risk of colon cancer development [17]. Recently, it was shown that higher apoAI and lower C reactive protein serum concentrations are independent predictors of overall survival in CRC [62]. In addition, lower serum apoAI levels were associated with a higher CRC stage and an enhanced systemic inflammatory response [63]. Moreover, post-chemotherapy increase in serum apoAI levels in association with improved survival was recorded [25]. Yet, it should be noted that the results of the Malmö Diet and Cancer Study do not support any significant relationship between serum apoAI levels and the risk of CRC development [64].

The principal role of apoAI is related to HDL maturation within RCT, and it was shown that the apoAI-driven uptake of cholesterol decreases the malignant features of cells in CRC tissue samples [65]. However, studies have implicated that apoAI possesses immunomodulatory and anti-inflammatory activities, too. Namely, it has been repeatedly reported that apoAI participates in the antiviral and antibacterial immune response, as well as in the suppression of chronic inflammation [66]. A bulk of evidence indicates that the antitumor activity of apoAI in various malignancies is partially related to its effects on immunity [66]. Indeed, it was recently shown that serum HDL-C levels correlate with the specific immune signature of cancerous tissue in CRC patients, in particular with CD3 and CD8 markers, implying that high HDL-C and apoAI levels might be associated with enhanced recruitment and activity of CD3+ and CD8+ T cells, which exhibit antitumor activities [29]. In addition, in vitro studies have demonstrated that apoAI mimetics inhibit the proliferation, migration, and invasiveness of human colon adenocarcinoma cell lines [67], as well as diminish the viability and proliferation of murine colon adenocarcinoma cells [67].

The fact that the *APOA1* gene is highly expressed and, consequently, apoAI highly abundant in the colon mucosa further strengths the hypothesis of potential contribution of apoAI to decrease of risk of CRC development. Evidence indicates apoAI-mediated amelioration of colitis and reduced susceptibility to colitis-associated carcinogenesis in mice [68]. Similar results were shown in an inflammatory bowel disease model [69], thus implicating the possible use of apoAI mimetics in the prevention and therapy of CRC. A recent study [70] demonstrated the synergistic inhibitory effects of apoAI and apoAI-binding protein (AIBP) on the migration and invasiveness of colon cancer cells, as well as on tumor-induced angiogenesis. The mentioned effects are reportedly achieved through promotion of cholesterol efflux and consequent suppression of the lipid raft-mediated signaling mechanism [70]. Taken altogether, the available evidence implies a plausible causal relationship between apoAI and a lower risk of CRC onset and progression.

### 3.2. ApoM

ApoM is an HDL-associated protein, but in contrast to the highly abundant apoAI, this apolipoprotein accounts for approximately 5% of HDL’s structure [71]. However, in spite of its minor presence within HDL particles, significant metabolic effects of apoM are reported. Namely, studies have shown that apoM enhances cholesterol efflux, as well as the anti-oxidative and anti-inflammatory functions of HDL [71,72,73,74,75,76], suggesting its beneficial effects in protection against cancer development.

An intriguing link between apoM and malignant diseases is related to the specific interaction of apoM with sphyngosine-1-phosphate (S1P). ApoM serves as a chaperone for S1P on HDL particles, and the biological activity of S1P depends on its relationship with apoM/HDL [77]. Apart from the well-understood role of S1P/apoM in cardiometabolic alterations [77], this interaction might be important for CRC development as well. In a review paper, Bao et al. [78] summarized the available findings regarding the contribution of the sphingosine kinase 1 (Sphk1)/S1P signaling pathway in CRC. Sphk1 is responsible for phosphorylation of sphingosine and production of S1P, and it was shown that Sphk1/S1P signaling can induce colorectal carcinogenesis through activation of the interleukin-6/signal transducer and activator of transcription (STAT)-3/protein kinase B pathway [78]. Moreover, S1P exhibits anti-apoptotic and angiogenic activity [79,80,81], which is an additional contributory mechanism for malignant transformation of cells. Thus, the existence of apoM/S1P interaction on HDL changes the prevailing perception of HDL as an antitumor biomolecule. However, the role of the HDL/apoM/S1P axis in CRC should be cautiously interpreted, since many of its aspects are still insufficiently explored. S1P exhibits its effects through interaction with five different types of receptors and receptor-independently, causing distinct metabolic effects, although the available evidence, in general, supports its cancer-promoting activity [82]. A study by Liang et al. [83] showed that in contrast to the tumor-promoting activity of Sphk1 [84], a lack of sphignosine kinase 2 (Sphk2) is associated with enhanced expression of Sphk1 and S1P receptor 1 and subsequent colitis-associated cancer development. Yet, other studies have reported positive associations between Sphk2 and colorectal carcinogenesis [85,86], thus implying the need for further investigation. It is noteworthy that atherosclerosis studies have revealed that the pro- or anti-atherosclerotic properties of S1P depend on whether it is associated with HDL/apoM or its secondary carrier, albumin [87]. Whether the same is true for colorectal carcinogenesis remains to be elucidated.

Regardless of its associations with S1P, evidence indicates the beneficial role of apoM in various malignancies [88,89]. Importantly, it was shown that apoM increases the expression of vitamin D receptor in CRC cells [90]. Appreciating the anti-proliferative, pro-apoptotic, and pro-differentiating effects of vitamin D [91], the above-mentioned findings [90] open a novel perspective for investigation of the role of HDL/apoM in carcinogenesis.

### 3.3. PON1

Two of three enzymes of the paraoxonase family, PON1 and PON3, reside on HDL particles, but PON1 is a far more studied member. In contrast to PON3, which is located both intracellularly and on HDL, PON1 is predominantly associated with HDL [92]. It is considered that the principal physiological role of PON1 is to protect both LDL and cell membranes from oxidation [93]. It is the anti-oxidant capacity that brought this enzyme into the focus of investigations regarding its possible anticancer properties. Since elevated oxidative stress is inherent to cancer development, a lack of PON1 activity could be expected in this condition. Lower serum PON1 activities were found in various malignancies, including CRC [94], although the opposite results were also noted but in a rather small cohort [95]. Our results also showed diminished PON1 paraoxonase and arylesterase activities in patients with CRC, alongside an elevated pro-oxidant/anti-oxidant balance [96].

An additional piece in the puzzling relationship between HDL/PON1 and CRC might be related to PON1′s association with eNOS and NO production. Namely, it is known that PON1 enhances HDL-mediated activation of eNOS and production of NO [97]. It has also been shown that eNOS can contribute to the development of CRC, primarily through the stimulation of angiogenesis [98,99], so the supposed protective role of PON1 in CRC development might be put in question by these findings.

It is still unclear whether the observed decrease in PON1 activity in CRC patients is causally related to cancer development or whether it is merely a reflection of cancer-induced metabolic changes. An observational study of the most studied PON1 Q192R and L55M gene polymorphisms indicated that QQ and LL genotypes are more prevalent in CRC patients, thus implying the possible contribution of specific PON1 genetic variants to CRC development [100]. In contrast, a more recent study by Abudayyak et al. [101] showed no differences in Q192R and L55M genotype frequencies between CRC patients and controls, similar to Van Der Logt et al.’s findings in a larger cohort of 365 cases and 354 controls [102]. However, it was also demonstrated that additional polymorphism of the PON1 gene rs3917538 is positively associated with overall survival but also with the response to 5-fluorouracil-based therapy [103], and such findings lay the groundwork for more extensive exploration of PON1 in CRC. Finally, it should be mentioned that several recent studies have reported the potential significance of PON1 as an additional biomarker of CRC [104,105].

Another significant aspect should not be neglected when discussing the potential contribution of PON1 in preventing CRC development. Namely, it has been firmly established that HDL is more than just a PON1 carrier and that structural alterations in HDL can profoundly affect PON1 activity [93,106]. PON1 is preferably associated with smaller HDL 3 particles, and a recent pilot study demonstrated that a loss of PON1 activity is associated with a decreased prevalence of small HDL particles during the hyperacute phase following surgical treatment of CRC [8]. Moreover, since apoAI is essential for the association of PON1 and HDL [106], it is reasonable to assume that any changes that affect apoAI in CRC patients should reflect on PON1 activity, too. As a confirmation, it was shown that PON1 activity decreases as the serum amyloid A (SAA) level increases during the acute phase after surgical treatment of CRC [8], which could be explained by the fact that SAA readily replaces apoAI within HDL particles in the inflammatory state. In addition, it was demonstrated that myeloperoxidase (MPO), an enzyme produced by neutrophils, monocytes, and macrophages, associates with HDL and PON1 in a ternary complex, wherein MPO and PON1 reduce each other’s activities [106]. As CRC is strongly associated with inflammation, there is evidence implying an increased number of MPO-positive cells in the normal colorectal mucosa of CRC patients [107]. It is noteworthy that an increased level of systemic oxidative stress was found in healthy subjects with higher expression of MPO-positive cells in the colonic mucosa [108], which could affect PON1 activity in healthy individuals as in CRC patients. However, PON1-induced mitigation of MPO activity might lead to reduced production of free radicals and acrolein, which is a factor associated with colorectal carcinogenesis [109]. Therefore, this topic deserves further investigation.

### 3.4. LCAT

LCAT catalyzes esterification of free cholesterol within HDL, thereby promoting maturation of HDL particles [110]. Albeit this protein is not largely explored in CRC, there is evidence of its downregulation in various other types of cancer, including hepatocellular carcinoma [111,112,113], ovarian cancer [114], and non-Hodgkin lymphoma [115]. Accordingly, our findings indicated reduced LCAT activity in patients with CRC [96]. Interestingly, it was shown that the levels of free cholesterol decrease in the serum of CRC patients when compared to healthy individuals, whereas they increase in cancerous tissue when compared to the adjacent mucosa [19]. Since free cholesterol is a substrate for LCAT, such alterations might be related to the observed decreased serum LCAT activity, but it remains to be elucidated whether diminished LCAT activity is a cause or a consequence of changed free cholesterol levels. Moreover, activation of LCAT is dependent on apoAI [116]; thus, abnormal activity of this enzyme might arise as a result of lowered apoAI levels in CRC patients. In addition, reduced liver function, which is readily seen in advanced CRC [117], might be a reason for lower LCAT concentration and activity, since LCAT is primarily synthesized by the liver [110]. Previous studies have demonstrated that hypermethylation is associated with lower LCAT gene expression in hepatocellular carcinoma [111,112]. According to our knowledge, similar studies have still not been performed in CRC.

### 3.5. CETP

CETP, as a transfer protein that enables cholesteryl ester transfer between HDL and -triglyceride-rich lipoproteins, is a well-known contributor to cardiovascular diseases [118], but its function in cancer is still insufficiently investigated. Apart from promoting lipoproteins’ cholesterol exchange, CETP also participates in cholesterol uptake by various cells, whereas intracellular CETP enhances cholesterol transport and storage within cells [119,120]. It was shown that CETP facilitates cholesteryl ester transport from HDL to HepG2 liver tumor cells [121]. Huang et al. [122] demonstrated that downregulation of CETP expression led to decreased uptake of cholesteryl esters from HDL by HepG2 cells via scavenger receptor class B type I (SR-BI). Knowing that malignant cells have increased demands for cholesterol, such CETP-enhanced cholesterol enrichment might contribute to their viability and malignant potential. It was demonstrated that CETP contributes to better survival of breast cancer cells and promotes their resistance to apoptosis [123]. However, it should be noted that recent Mendelian randomization studies have revealed opposite findings, by demonstrating the association of CETP downregulation with an increased risk of breast cancer [124,125]. Similarly, decreased *CETP* gene expression was found in gallbladder cancer tissue [126]. In our study of lipid mediators in CRC [96], we demonstrated increased CETP activity in CRC patients, in spite of a decreased plasma concentration of this protein, which could be linked to the enhanced uptake of cholesterol by CRC cells due to their increased need. Moreover, the CETP protein level was singled out as a potential biomarker of CRC [96]. Such results warrant further investigations of CETP-mediated contributions to various types of cancer, including CRC.

## 4. Genetic Regulation of HDL Metabolism in CRC

Keeping in mind the overall contribution of inheritance to CRC development [127], the complex genetic background of HDL particles should not be neglected in consideration of their role in CRC. Since numerous mediators are included in proper HDL functioning, genetic factors responsible for their regulation might be involved in the etiopathogenesis of various disorders, including cancer. As will be presented herein, the role of genetic and epigenetic changes in significant HDL modulators has been analyzed in CRC. Investigated genetic contributors to the disturbed HDL homeostasis in CRC and their presumable clinical potential in CRC are summarized in Table 1.

### 4.1. ABC Transporters

Two ABC transporters, ABCA1 and ABCG1, being essential components of RCT, are important contributors to the processes of HDL biogenesis and particle maturation. Liver X receptors α/β (LXRα/β) agonists, as well as ligands for peroxisome proliferator-activated receptors (PPARs), are recognized as the main regulators of *ABCA1* and *ABCG1* gene expression in macrophages, hepatocytes, fibroblasts, and intestinal cells, while the transcription factor retinoic-acid-related orphan receptor α (RORα) regulates the expression of these transporters in intraabdominal adipose tissue [128,129,130,131]. In addition, apart from ABCA1 and ABCG1 regulation of HDL metabolism, their corresponding subfamilies are involved in the development of resistance to chemotherapeutics [132]. Although several different ABC family members have been singled out due to their individual contribution to cancer progression and failure of therapy, due to the plethora of transporters’ effects, investigations have focused on the entire transcripts’ profiles as well. Hlavata et al. showed that altered ABC gene expression levels represent useful CRC prognostic tools [133]. Even though lower mRNA levels of *ABCA1* and *ABCG1* were reported, these particular constitutes of the ABC family did not exhibit a significant potential to be used as CRC prognostic markers. Conversely, findings of Aguirre-Portolés et al. [65] supported the relevance of *ABCA1* gene alterations in CRC, pointing toward upregulated *ABCA1* gene expression as a marker of CRC invasiveness and overall survival. The same authors further reported a causal relationship between *ABCA1* overexpression and increased invasiveness of CRC cells due to altered epithelial-to-mesenchymal transition (EMT) through E-cadherin- and vimentin-mediated effects [65]. This finding is in line with our recent evaluation of E-cadherin as a predictor of CRC’s onset, with a high diagnostic potential [134]. Another study, the ColoLipidGene signature, investigated the relationship between lipid-metabolism-associated genes and CRC progression in patients with stage II of the disease [135,136]. Later, the prognostic potential was broadened to all stages of CRC [136]. Namely, *ABCA1* gene expression was clustered with Acyl-CoA synthetase 1 (*ACSL1*), 1-acylglycerol-3-phosphate O-acyltransferase 1(*AGPAT1*), and stearoyl-CoA-desaturase 1 (*SCD*) in a fingerprint, which was singled out as a prognostic classifier for the 3-year DFS [135]. Overexpression of *ABCA1*, *AGPAT1*, and *ACSL1* is associated with worse CRC outcomes, while *ABCA1* and *AGPAT1* are emphasized as predictors of a higher risk of CRC recurrence [136]. Interestingly, the authors [136] also revealed the association between the non-synonymous polymorphism rs2230808, located on the *ABCA1* gene, and its mRNA levels. Patients carrying at least one copy of the minor allele T had higher *ABCA1* mRNA levels than those homozygous for the major allele CC [136]. Considering polymorphisms as one of the most important sources of genetic variability, this combination of genotyping and transcriptome analyses might provide a highly anticipated personalized approach to cancer diagnostics and ensure complete information about the overall protein synthesis and, subsequently, its function. Therefore, these types of studies are highly desirable.

Although more data refer to ABCA1, the role of ABCG1 in CRC development should not be underestimated. This transporter has been associated with CRC cancer progression, since its increased gene expression was found in aggregative metastatic colon cancer cells [137]. The same study suggested that ABCG1 could be used as a potential therapeutic target, considering that its depletion leads to the accumulation of extracellular vesicles, which results in tumor regression [137]. Furthermore, ABCG1-mediated efflux of cholesterol and phospholipids was proposed as a protective mechanism that protects cancer cells against toxicity caused by over-accumulation of lipids [137]. A study by Po et al. also supported the relevance of *ABCG1* gene expression alterations in CRC [132]. Namely, the authors reported increased *ABCG1* mRNA levels as a predictor of poor disease outcomes [132].

Bearing in mind the epigenetic modulations of mRNA, a number of studies have bene devoted to small interfering RNA (siRNA) and microRNA (miRNA) species as potential therapeutic agents in CRC [137,138]. As the majority of studies imply that overexpression of *ABCA1* or *ABCG1* is related to poor CRC prognosis, these genes might represent targets for epigenetic therapy. However, a study conducted by Bi et al. [139] showed that overexpression of miR-183 results in a reduction in *ABCA1* gene expression, which leads to enhanced proliferation and attenuated apoptosis of colon tumor cells. Thus, in contrast to previously cited findings, ABCA1 is proposed as a potential colon cancer suppressor, while miR-183 is proposed as an oncogene [139]. Taken altogether, the involvement of genetic alterations of ABCA1 and ABCG1 in CRC development is still unclear, and further studies are necessary.

ABCG5 and ABCG8 transporters, which are predominantly localized in the gall bladder, intestine, and liver [140], are also involved in cholesterol metabolism. It has been shown that oxysterol-sensing LXR nuclear receptors represent one of the main regulators of these transporters’ expression levels, while GATA4 and GATA6 transcription factors, together with hepatocyte nuclear factor 4α, induce their transcription in a cooperative manner [141,142]. Since ABCG5 and ABCG8 transporters are important for biliary and intestinal sterols’ elimination, alterations in their function affect the total cholesterol metabolism, consequently influencing HDL homeostasis. While alterations in the ABCG5/8 genetic locus (sitosterolemia locus (STSL)) have been widely associated with sitosterolemia, there is a question of whether changes in ABCG5 and ABCG8 transporters might cause a higher susceptibility to other diseases such as cancer. According to the genome-wide association study of the European population, disturbances in lipid levels are related to *ABCG5* polymorphic variants [143]. The authors reported a causal relationship of the single-nucleotide polymorphism (SNP) *ABCG5* rs6756629 with total cholesteroland LDL-C [143]. Meanwhile, in a population of Puerto Ricans, lower HDL-C concentrations were obtained in C allele carriers of the *ABCG5* i7892T>C (rs4131229) polymorphism in comparison to TT subjects [144]. Lower HDL-C concentrations were also reported in the minor allele carriers of *ABCG8* polymorphisms, *ABCG8* 5U145 A>C (rs3806471) and Tyr54CysA>G (rs4148211), as well as in major allele homozygotes of the *ABCG8* Thr400LysC>A (rs4148217) sequence variant [144]. A review of the available literature also showed that associations between different SNPs of *ABCG5* or *ABCG8* and metabolic status traits (such as lipids alterations) were mostly explored in subjects with gallstone disease or gallbladder cancer due to the transporters’ specific localization and their prominent role in the efflux of sterols. However, to the best of our knowledge, there are no studies regarding the association of these SNPs with CRC risk. Namely, we found only two studies that reported transcriptomic data for *ABCG5* and *ABCG8* in CRC. Hlavata et al. [133] showed that there were no differences in *ABCG5* and *ABCG8* mRNA levels between tumor and control tissues, with transcript levels decreasing from the colon to the rectum, while the other study provided evidence of reduced survival time in lymph-node-negative CRC patients with ABCG5-positive tumor buds [145]. Taken altogether, this could represent a valuable research topic for studies of metabolic types of malignancies, such as CRC, with evident alterations in HDL-C.

### 4.2. Scavenger Receptor Class B, Type 1

Scavenger receptor class B, type 1 (SR-B1, coded by the *SCARB1* gene) represents another important element in maintaining HDL homeostasis. Given its role in the regulation of cellular uptake of cholesterol from HDL, genetic alterations of this receptor may affect multiple functions and processes [146]. The highest *SCARB1* mRNA levels were found in steroidogenic tissues and the liver [146,147]. Many factors that regulate lipid homeostasis affect *SCARB1* gene expression as well [146]. Taking into account the high demands of malignant cells for cholesterol, SR-B1 might be considered a mediator of cholesterol uptake in cancer tissues [148]. The most extensively studied contribution of SR-B1 to cancer development is in prostate and breast malignancies [148,149]. However, while inspecting HDL nanoparticles as siRNA therapeutic loads, Shahzad et al. discovered that CRC cell lines have increased expression of SR-B1 as well [150]. Sharma et al. [151] did not find any statistically significant difference in LDL receptor (*LDLR*), *SCARB1*, and *ABCA1* mRNA levels when comparing CRC tumor tissue with the normal adjacent mucosa. In contrast, when TCGA Colon Adenocarcinoma and Rectal Adenocarcinoma datasets were used, higher mRNA levels were obtained for *LDLR* and *SCARB1*, while *ABCA1* was downregulated [151].

**Table 1:** Relevant gene expressions and single-nucleotide polymorphisms (SNPs) related to HDL homeostasis in CRC.

Sources	Gene Expression/SNP Findings	Possible Clinical Significance
Hlavata et al. [133]	Decreased ATP-binding cassette transporter A1 (*ABCA1*) and *ABCG1* mRNA levels in CRC tumor tissue No differences in *ABCG5* and *ABCG8* transcript levels between tumor and control tissues Decrease in *ABCG5* and *ABCG8* transcript levels from colon to rectum	Assumed prognostic potential of ABC transporters
Aguirre-Portolés et al. [65]	Increased *ABCA1* gene expression in advanced stages of CRC	Marker of CRC invasiveness and overall survival
Vargas et al. [135] Fernández et al. [136]	Increased *ABCA1* gene expression in a prognostic fingerprint (ColoLipidGene signature), primarily used for stage II of CRC and later extended to all stages of CRC Non-synonymous *ABCA1* polymorphism (rs2230808) association with *ABCA1* mRNA levels	Prognostic marker (emphasized as the main carrier of ColoLipidGene signature potential)
Namba et al. [137]	Increased *ABCG1* gene expression in aggregative metastatic colon cancer cells Decreased viability of tumoroids caused by ABCG1 depletion	Possible therapeutic target Prognostic marker for patients with CRC
Po et al. [132]	Increased *ABCG1* gene expression in CRC tissue Increased *ABCG1* expression related to worse disease prognosis	Prognostic biomarker
Bi et al. [139]	MicroRNA (miR)-183 downregulating *ABCA1* gene and protein expression in colon cancer cells Silencing of *ABCA1* gene increasing proliferating capacity and inhibiting tumor cells’ apoptosis	*ABCA1* singled out as a tumor-suppressive gene in colon cancer miR-183 named as a possible predictive biomarker of advanced aggressive forms of CRC
Hostettler et al. [145]	Poor survival time obtained in patients with ABCG5-positive tumor buds	Prognostic biomarker
Shahzad et al. [150]	Increased expression of scavenger receptor class B, type 1 (SR-B1) in breast, colorectal, pancreatic, and ovarian cancer cell lines	SR-B1 representing potential therapeutic targets for delivery of small interfering RNA (siRNA)-based therapeutic HDL’s payloads
Sharma et al. [151]	Inconsistent results regarding low-density lipoprotein receptor (*LDLR*), *SCARB1*, and *ABCA1* mRNA levels between experimental and in silico analysis of colorectal tissue and adjacent/normal mucosa TCGA cohort: increased *LDLR* and *SCARB1* expression but downregulated *ABCA1*	Evaluation of diagnostic and prognostic potential of biomarkers included in total cholesterol homeostasis

### 4.3. LCAT and CETP

Available data showed that mRNA levels of *LCAT* have been mostly explored within the prognostic models of hepatocellular carcinoma [111,112,152]. It has been shown that *LCAT* represents a DNA-methylation-driven gene, implying that environmental factors can alter *LCAT* mRNA levels through epigenetic modifications [111,112]. In addition, a study by Johnson et al. [124] pointed toward positive relationships between three genetic loci related to HDL homeostasis and breast carcinoma risk: *ABCA1*, *APOE-APOC1-APOC4-APOC2*, and *CETP*. However, these types of associations are largely unexplored in CRC. It is also noteworthy that SNPs related to *LCAT* and *CETP* are well investigated in various pathological conditions. The *CETP* rs708272 or Taq IB polymorphism is well-known for its strong association with HDL-C levels, while also being in high concordance with the CETP -629 A/C polymorphism [153]. The Taq IB polymorphism is reportedly associated with metabolic syndrome [154,155,156], and knowing that this syndrome is a risk factor for CRC development, this could be an interesting subject for future research. Although the relationship between metabolic syndrome, obesity, and CRC has been widely proven [157,158], a challenge for future researchers is to examine the significance of the most prevalent *CETP* and *LCAT* polymorphisms for the onset of CRC. During an extensive literature survey, we found two studies that established the connection of *CETP* rs708272 genotypes with the risk of gallbladder cancer as well as with the risk of pituitary adenoma [159,160], but none of these were related to CRC. Exploration of gene–gene and gene–environmental interactions demonstrated that *APOA1*, *ABCA1*, and *LCAT* polymorphisms significantly contribute to dyslipidemia development [161]. This type of analysis supports the significance of genetic factors in the etiology of lipid disorders, which are also included in CRC etiology. Given the abundance of research dealing with genetic alterations of HDL in various diseases, including several types of cancer, this subject should be more extensively investigated with regard to CRC.

## 5. HDL and Cholesterol Trafficking in CRC

Changes in the overall cholesterol metabolism have been observed in malignant diseases. As numerous studies have shown, the mevalonate pathway of cholesterol synthesis is upregulated in a number of cancers (leukemia, lymphoma, multiple myeloma, breast, lung, liver, pancreatic, esophageal, and prostate cancers) [162,163]. However, even though increased cholesterol concentrations represent a risk factor for CRC [164], low serum total cholesterol concentrations have been observed in these patients. It is assumed that progression of premalignant lesions can lead to a significant reduction in circulating cholesterol levels. In fact, a study by Broitman et al. showed that low cholesterol concentrations occur 10 years prior to colon cancer diagnosis [165]. A possible reason for such depletion in plasma cholesterol might be its increased cellular uptake, which is necessary for the growth and development of premalignant and malignant tissues [165,166].

Knowing that cholesterol is immanent for malignant cell survival, HDL-mediated cholesterol trafficking could play a significant role in tumor development [167]. Such assumption brings a novel perspective to the consideration of the role of HDL in malignant diseases. Indeed, several studies have shown that HDL particles can stimulate the growth of breast cancer cells in vitro, as well as increase the aggressiveness of malignant tumors in mice [168,169]. The addition of HDL to cell cultures induced the proliferation and migration of androgen-independent prostate cancer cells [170]. These findings are consistent with studies that have shown increased SR-B1 expression in cancer cells, suggesting that during carcinogenesis, tumor cells use HDL to meet their needs for cholesterol [171]. It has been shown that SR-B1 expression is directly proportional to tumor aggressiveness and that tumor progression is reduced by a decrease in SR-B1 activity and expression [169,172]. Diminished HDL-C levels, which are characteristic of cancer, are accompanied by increased expression of SR-B1 [146]. Still, it remains to be investigated to what extent the increased functionality of SR-B1 contributes to the observed lowering of plasma HDL-C levels. Results of a study conducted by our research group also demonstrated lower HDL-C levels in CRC patients [173]. However, we observed that the relative content of cholesterol precursors on HDL particles is higher when compared to healthy controls (unpublished data). Recent studies have shown that during intensive cholesterol synthesis, precursors of cholesterol are also taken up by HDL particles [174]. In light of this, our findings may indicate that even though there is a decrease in HDL-C levels in CRC patients, cholesterol is increasingly produced in peripheral tissues and transported by HDL to malignant cells in order to be taken up via SR-B1. In line with this, several studies have observed that high expression of SR-B1 can cause increased growth of malignant cells incubated with HDL, leading to the conclusion that larger cholesterol ester-enriched HDL particles, which represent a better substrate for SR-B1-mediated cholesterol influx, may support cancer cell growth [175].

As mentioned before, not only cholesterol but also a range of its metabolites are transported via HDL [174]. Oxysterols are the most important intermediates in the biotransformation of cholesterol. The source of exogenous oxysterols is cholesterol-rich food [176]. Endogenous oxysterols are formed as reaction products catalyzed by CYP450-dependent or independent hydroxylases [177]. Oxysterols, acting as ligands of LXRα (NR1H3) and LXRβ (NR1H2), can regulate the transcription of specific genes [178]. Given that LXRα is involved in the regulation of ABCA1 and ABCG1 proteins and that LXRs also seem to play a role in the regulation of CETP [166], it can be assumed that oxysterols also affect HDL metabolism.

In contrast, previous results have shown that oxysterols, but not cholesterol per se, have inflammatory and degenerative potential and increase the risk of developing CRC [176]. Oxysterols participate in the modulation of inflammatory and apoptotic cell signaling cascades, thus affecting the growth and survival of cancer cells [179]. Studies have suggested that oxysterols are involved in various steps of carcinogenesis and the progression of CRC by impairing intestinal barrier integrity and immune escape action [166,176,177,178]. Swan et al. demonstrated the overexpression of enzymes involved in oxysterol synthesis in CRC tissues, which is also associated with poorer prognosis [179]. In light of this, the role of HDL in oxysterols’ transport and distribution to malignant cells should not be neglected. Indeed, it has been shown that 27-hydroxycholesterol (27-OHC) is abundantly transported via HDL [175]. A study conducted by Rosin et al. [180] demonstrated a higher concentration of 27-OHC in malignant tissue compared to the surrounding mucosa in advanced stages of CRC. In the same study, the authors demonstrated that high concentrations of 27-OHC increase the release of pro-inflammatory cytokines in the Caco-2 cell line [180]. In contrast, a study examining the effect of different concentrations of 27-OHC on two adenocarcinoma cell lines showed that 27-OHC significantly reduces the activation of AKT protein kinase involved in cancer pathogenesis in Caco-2 but not in colorectal adenocarcinoma SW620 cell lines [176]. Thus, the involvement of oxysterols in the etiopathology of CRC should be further examined, alongside the role of HDL in this process.

## 6. Possible Therapeutic Implications

Undoubtedly, an important role of lipoproteins in carcinogenesis opens intriguing possibilities when it comes to cancer therapy. Statins were first introduced in studies as potential cancer-preventive agents or as an addition to cancer therapy, since they reduce cholesterol synthesis and its availability to malignant tissue. Moreover, statins exhibit other pleiotropic effects, such as anti-inflammatory and anti-oxidative properties. Evidence of the effect of statin treatment on CRC risk is rather conflicting. A recent study by Lee et al. [181] showed that statin therapy is associated with reduced CRC risk, whereas Shah et al. [182] revealed that statin therapy does not significantly modify the risk of CRC development in patients with inflammatory bowel disease. It was also observed that patients receiving statins prior to and during CRC treatment may have good overall and cancer-specific survival rates [183].

Additionally, many other studies have analyzed different molecules involved in the metabolism of cholesterol and HDL particles as therapeutic targets in CRC treatment. It has been postulated that membrane lipid rafts play a crucial role in cancer initiation, development, and progression [184]. Since lipid rafts represent cholesterol-rich parts of the plasma membrane, direct or indirect modulation of their cholesterol content may be of potential interest for anticancer therapy. Among others, apoAI, which interacts with the plasma membrane via ABC family transporters, and SR-B1 have vast potential as therapeutic agents in malignant diseases [167]. A recent study by Mao et al. [185] showed that AIBP cooperates with HDL, thus leading to the restriction of endothelial cell migration and angiogenesis, through enhancement of cholesterol efflux and reduction in the lipid raft content. A synergy between AIBP and apoAI/HDL seems to be crucial in regulating intracellular cholesterol levels. Results of a recent study by Zhang et al. [70] showed that AIBP and apoAI inhibit intestinal tumor growth, migration, invasion, metastasis, and angiogenesis. Treatment consisting solely of apoAI induces discrete cholesterol efflux activity, which insufficiently alters cellular functions. In contrast, AIBP cannot trigger cholesterol efflux in the absence of apoAI [70].

The bromodomain extraterminal (BET) family of proteins includes co-activators of RNA polymerase II, and small molecules targeting BET proteins (BET inhibitors) preferentially suppress the transcription of cancer-promoting genes [186]. RVX-208 or apabetalone was originally discovered during the screening for apoAI mRNA inducers in cultured hepatocytes [187] and currently represents the most promising BET inhibitor in clinical studies. A study by Aguirre-Portolés et al. [65] showed that proliferation rates were slower upon apabetalone treatment of both colorectal adenocarcinoma DLD1 and Caco-2 cell lines. Additionally, it was shown that apabetalone could revert the EMT phenotype present in cells that overexpress ABCA1. Most ATP-binding cassette transporters act as substrate exporters [188], but Yamauchi et al. [174] showed that ABCA1 could mediate the bidirectional substrate flux, which means that cholesterol can also be taken up by cells via this transporter. This is especially important for malignant cells, considering their extensive needs for cholesterol. The treatment of overexpressing ABCA1 cells with apabetalone resulted in decreased levels of ABCA1 comparable to those in healthy cells. In addition, it was observed that through apoAI upregulation, apabetalone diminishes the malignant potential of CRC cells [65].

Accumulating evidence suggests that apoAI also targets tumor-promoted inflammation. The proliferation, invasiveness, and migration of human ABCA1-transfected colon adenocarcinoma cells can be inhibited by downregulation of cyclooxygenase 2 (COX-2), caused either by simultaneous transgenic overexpression of apoAI or by exogenous treatment with human recombinant apoAI [65]. Additionally, it was observed that lysophosphatidic acid (LPA), vascular endothelial growth factor (VEGF), and hypoxia-inducible factor α (HIF-1 α) levels were significantly reduced in apoAI mimetic peptide-treated mice [66]. One of the apoAI mimetic peptides is 4F, which has more potent anti-oxidant and anti-inflammatory properties than native apoAI [189]. Both D-4F, synthesized from D-amino acids, and L-4F, synthesized from L-amino acids, exhibit comparable in vitro and in vivo properties, and they both successfully inhibit CRC tumor growth in murine models [190,191]. The presence of 4F in HDL enables the transfer of oxidized lipids from LDL to HDL and their subsequent elimination via RCT [191]. A study by Cedó et al. [191] showed that D-4F, but not apoAI-containing HDL, significantly increases tumor latency, inhibits the development of tumors, and reduces plasma oxidized low-density lipoprotein (oxLDL) levels in mice, preventing an oxLDL-mediated proliferative response in human breast adenocarcinoma MCF-7 cells.

Another aspect of th epossible use of HDL in cancer therapy refers to specific targeting of malignant cells. Namely, since non-malignant tissue shows significantly lower expression of SR-B1 compared to malignant tissue, it is suggested that HDL particles can be used as drug delivery systems and can have beneficial effects not only on the restriction of tumor growth but also on reduction in chemotherapy-induced cytotoxicity [167]. In recent years, HDL-based nanoparticles, as therapeutic delivery systems, have been developed to target tumor cells by taking advantage of their unique property to overexpress SR-B1 [192].

Reconstituted HDL (rHDL) and synthetic HDL (sHDL) represent HDL-mimicking particles derived from cholesterol-free HDL particles and apoAI, or short synthetic apoA-I mimetic peptides in complexes with phospholipids [193]. As such, they can serve as cholesterol efflux vehicles and deplete cells from excess cholesterol, target SR-B1-expressing cells, or act as drug delivery systems for nucleic-acid-based therapies, proteins, and other anticancer therapeutics, as well as for labeling and diagnostic imaging of cancer cells [34,194]. Recently, sHDL nanoparticles with gold nanoparticles that target SR-B1 were designed as a size- and shape-restrictive template for lymphoma starvation therapy. After treating mice with these nanoparticles, tumor growth and metastatic tumor burden significantly reduced and the survival rate improved [195]. Such specific targeting of SR-B1 promotes cholesterol efflux, limits cholesterol delivery, and induces cancer cells’ starvation and consequent apoptosis [196]. Shahzad et al. managed to achieve rHDL-based targeted delivery of small interfering RNA by taking advantage of SR-B1 overexpression in CRC cells [150]. A current goal is to further enhance rHDL nanoparticles’ specificity to target tumor cells. With this in mind, Ye et al. used L-4F to enhance binding between nanoparticles and tumor cells, and by this approach, a longer median survival rate was achieved [197]. Another interesting approach was using the 5A peptide in the synthesis of docetaxel (DTX)-loaded sHDL, which exhibited sustained drug release, high cytotoxicity directed toward MCF-7 breast cancer cells, and prolonged survival in mice [192].

All of the above findings highlight the promising potential of HDL-related therapy in the treatment of malignancies, including CRC. However, extensive clinical trials are needed in order to introduce this type of therapy into future therapeutic protocols.

## 7. Conclusions

In summary, the role of HDL during the onset and progression of CRC is still not fully understood. Although decreased serum HDL-C levels are typically seen in CRC patients, the complex structure and diverse functions of this remarkable lipoprotein, as well as its interactions with other biomolecules, prevent us from drawing definitive conclusions and point toward divergent pathways of its involvement in colorectal carcinogenesis. Importantly, further studies are needed to establish whether the association of HDL with CRC development is causal or merely a reflection of disturbed metabolic balance in this disease. The answer to this question should direct future investigations of HDL-based therapeutic approaches in CRC. In addition, one of the imperatives of precision medicine is to distinguish patients at higher risk of disease onset and progression in order to improve their survival. Therefore, identification of non-invasive prognostic markers for the recurrence of CRC represents an area of great importance. Keeping in mind that changes in HDL-C levels, HDL particle distribution, as well as HDL receptors and modulators have been consistently found in CRC patients, HDL and associated molecules should be considered as promising biomarkers of CRC. Future investigations in this field are warranted.

## Data Availability

Not applicable.
